# GEF-independent Ran activation shifts a fraction of the protein to the cytoplasm and promotes cell proliferation

**DOI:** 10.1186/s43556-020-00011-2

**Published:** 2020-12-30

**Authors:** Jinhan Zhou, Yuping Tan, Yuqing Zhang, Aiping Tong, Xiaofei Shen, Xiaodong Sun, Da Jia, Qingxiang Sun

**Affiliations:** 1 Department of Pathology, State Key Laboratory of Biotherapy and Cancer Centre, West China Hospital, Sichuan University and Collaborative Innovation Center of Biotherapy, Chengdu, 610041 China; 2 Key Laboratory of Birth Defects and Related Diseases of Women and Children, Department of Paediatrics, Division of Neurology, West China Second University Hospital, Sichuan University, Chengdu, 610041 China; 3 Department of Pharmacology, West China School of Basic Medical Sciences and Forensic Medicine, Sichuan University, Chengdu, 610041 China

**Keywords:** Small GTPases, Nuclear transport, GTP bias, Activation, Cancer mutations

## Abstract

Ran (Ras-related nuclear protein) plays several important roles in nucleo-cytoplasmic transport, mitotic spindle formation, nuclear envelope/nuclear pore complex assembly, and other functions in the cytoplasm, as well as in cellular transformation when switched on. Unlike other members of the GTPase superfamily, Ran binds more tightly to GDP than to GTP due to the presence of an auto-inhibitory C-terminal tail. Multiple missense mutations in the C-terminus of Ran occur in cancers, but their biological significance remains unclear. Here, the quantitative GDP/GTP binding preference of four engineered mutations with unstable C-termini was analyzed using a devised mant-GDP dissociation assay. The results showed that the impact of different C-terminal mutations depends on multiple factors. Although these mutants were more GTP-loaded in human cells, they were shown to be more cytoplasmic, and to support nuclear transport with minimally or partially reduced efficiency. Further, several Ran cancer mutants were compromised in autoinhibition, slightly more GTP-bound, more cytoplasmic, and enhanced the proliferation of A549 and HeLa cells in vitro. Thus, our work reveals a new route of Ran activation independent of guanine nucleotide exchange factor (GEF), which may account for the hyper-proliferation induced by Ran cancer mutations.

## Introduction

Ran (Ras-related nuclear) protein is a member of the Ras superfamily of small GTPases. Like other GTPases, Ran switches between GDP-bound (inactive) and GTP-bound (active) states. The guanine nucleotide exchange factor (GEF) RCC1 (Regulator of Chromosome Condensation 1), which is chromatin-bound, increases the nucleotide exchange rate and charges Ran with GTP in the presence of abundant cellular GTP [1]. On the other hand, the GTPase activating protein (GAP) RanGAP, which is located on the cytoplasm face of nuclear pore complex (NPC), increases the rate of GTP hydrolysis on Ran [2]. The restricted localization of RCC1 and RanGAP creates a steep gradient of Ran-nucleotide: Ran is predominantly GTP-bound in the nucleus and GDP-bound in the cytoplasm [3].

Ran is well-studied for its role in nucleo-cytoplasmic transport [4, 5]. In the nucleoplasm, RanGTP unloads a nuclear localization signal (NLS)-containing cargo from an importin (e.g. importin β1), or forms a trimeric nuclear export complex with a nuclear export signal (NES)-containing-cargo and an exportin (e.g. CRM1) [6]. In the cytoplasm, the different Ran complexes are disassembled by RanGAP-mediated RanGTP hydrolysis with the help of Ran-binding protein 1 or 2 (RanBP1 or RanBP2) [7]. RanGDP is recycled back to the nucleus by nuclear transport factor 2 (NTF2) [8].

Besides performing this nuclear transport function, Ran is also involved in mitotic spindle formation, nuclear envelope/nuclear pore complex assembly, and diverse other functions in the cytoplasm [9, 10]. Ran is overexpressed in a few cancers, and its activation - which in this work refers to being more GTP-bound rather than enzymatically active - is involved in cell proliferation, metastasis, and cellular transformation [11–14]. Importantly, inhibition of Ran activation using an anti-RCC1 peptide demonstrates preferential cytotoxicity against breast cancer cells [12, 15]. Recently, Ran was shown to orchestrate ovarian cancer cell invasion through the stabilization of RhoA [16].

Unlike other Ras superfamily proteins, Ran contains a unique C-terminal tail that packs against its G-domain, probably accounting for its ten-fold lower affinity for GTP compared with GDP [17]. The C-terminal region is also critical for the binding of the important effectors RanBP1 and RanBP2 [7]. Multiple missense mutations in the C-terminal tail of Ran exist in the COSMIC and cBioPortal cancer database servers, but the biological significance is unclear. To understand the impact of C-terminal mutations, we designed four mutations that disrupt the interaction between the C-tail and the G domain. The designed mutants and the cancer mutants, were purified and characterized regarding their GTP/GDP binding preference, interaction with cellular regulators, subcellular localization, and ability to support nuclear transport. The ability of cancer mutants to promote cell proliferation and regulate endogenous pathways was also tested.

## Results

### The designed C-tail disrupting (C-dis) mutants were more biased to bind GTP

**Fig. 1:**
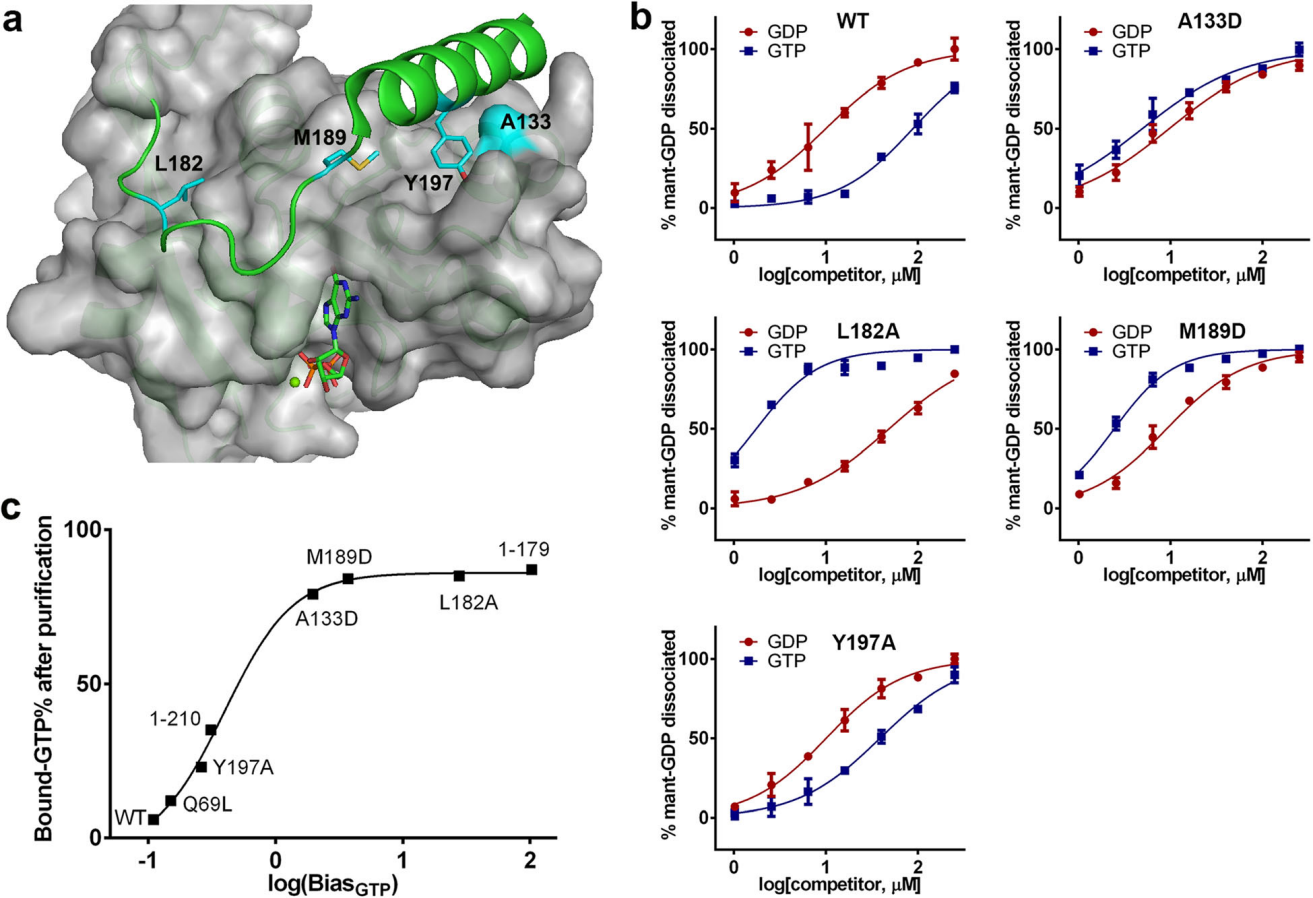
Higher GTP binding preference of Ran C-dis mutants. **a** Illustration of the mutation sites. Ran (3GJ0) is displayed as a green cartoon with the G-domain covered in a partially transparent grey surface. Four loci of mutagenesis (L182, M189, Y197, and A133) are shown as cyan surfaces. GDP is shown as sticks. **b** mant-GDP dissociation by different concentrations of GDP or GTP. Error bars represent standard deviations of triplicates. IC50 values are shown in Table 1. **c** Correlation between GTP% values (Supplementary Table 1) and GTP binding preference (Bias_GTP_ from Table 1, shown on a log scale). The Pearson correlation coefficient (CC) is 0.9. The line represents four-parameter logistic regression of the data

In order to study the function of C-terminal mutations, we designed four C-dis mutants (A133D, L182A, M189D, and Y197A) to disrupt C-tail binding to the G domain (Fig. 1a). We previously showed that the percent of bound-GTP (GTP%) was increased for these mutants after purification from *E. coli* (to be published, also in Supplementary Table 1). To know whether the increased level of GTP binding is due to increased preference to bind GTP over GDP, we compared the binding activity of Ran^WT^ (6% GTP) and Ran^M189D^ (84% GTP) in the presence of RCC1 and different ratios of GTP/GDP using a pull-down assay. Ran^M189D^ displayed at least ten-fold increased GTP/GDP relative affinity compared to Ran^WT^ (Supplementary Fig. 1).

To quantify the change of relative affinities, we devised a mant-GDP dissociation experiment wherein different proteins were first charged with mant-GDP in the presence of RCC1 (more than 50% mant-GDP charged, the ratio of bound GTP/GDP/mant-GDP was not determined), then the bound mant-GDP was dissociated by incubating with increasing concentrations of GDP or GTP (Fig. 1b). Two competitor-dissociation response curves can be plotted, from which the IC50 values, termed IC50_GDP_ and IC50_GTP_ can be determined. For one protein, GDP and GTP at their IC50 concentrations are equally effective at dissociating 50% of the bound mant-GDP under the same condition. The IC50_GDP_/IC50_GTP_ ratio, named as Bias_GTP_, thus represents a protein’s preference to bind GTP compared with GDP (Table 1). Bias_GTP_ value of one means that the protein is not biased to bind GDP vs. GTP. The larger the Bias_GTP_ number, the more biased the protein is to bind GTP. It should be noted that the Bias_GTP_ value may not be the real ratio of GTP/GDP affinity, but rather an approximation to the real ratio. Due to the pico-molar affinity of nucleotides and the instability of apo-Ran, it is rather difficult to obtain the real ratio [17]. Bias_GTP_ values are much easier and faster to acquire, yet are reproducible and appropriate when used to compare mutants.

**Table 1:** GDP/GTP binding preference (Bias_GTP_) of different Ran mutants. A larger value of Bias_GTP_ indicates that the protein more preferentially binds GTP. Bias_GTP_ value of 1 means binding GDP or GTP at equal affinity

Protein	Competitor	IC50 (μM)	95% Confid. Intervals (μM)	Bias_GTP_ (IC50_GDP_/IC50_GTP_)
WT	GDP	9.9	8.2 to 12.0	0.11
	GTP	87.8	78.4 to 98.3	
Q69L	GDP	11.4	10.3 to 12.6	0.15
	GTP	73.6	63.0 to 86.0	
1–179	GDP	395.7	326.2 to 480.0	102.9
	GTP	3.8	3.6 to 4.1	
1–210	GDP	18.3	15.4 to 21.6	0.31
	GTP	59.4	53.5 to 65.9	
A133D	GDP	9.6	8.2 to 11.2	1.96
	GTP	4.9	4.1 to 5.9	
L182A	GDP	48.5	43.6 to 54.0	27.7
	GTP	1.7	1.5 to 2.0	
M189D	GDP	9.1	7.9 to 10.4	3.71
	GTP	2.4	2.2 to 2.7	
Y197A	GDP	10.0	8.8 to 11.4	0.26
	GTP	38.5	33.7 to 44.1	
H30Y	GDP	13.8	12.3 to 15.5	0.20
	GTP	69.9	62.2 to 78.6	
V177A	GDP	14.0	12.5 to 15.6	0.25
	GTP	55.4	47.1 to 65.2	
M179I	GDP	13.9	12.1 to 16.1	0.20
	GTP	69.0	60.9 to 78.2	
P180L	GDP	11.3	10.3 to 12.4	0.57
	GTP	19.9	17.7 to 22.5	
A183T	GDP	8.1	7.5 to 8.8	0.40
	GTP	20.3	17.5 to 23.5	
P184S	GDP	8.3	7.4 to 9.3	0.20
	GTP	41.0	37.2 to 45.3	
V187A	GDP	10.2	9.2 to 11.3	0.22
	GTP	46.5	42.0 to 51.5	

The measured Bias_GTP_ value for Ran^WT^ was 0.11 (Fig. 1b), which agreed very well with a previous estimation [2], suggesting that the method was feasible. Ran^A133D^, Ran^L182A^, Ran^M189D^, and Ran^Y197A^ showed Bias_GTP_ values of 1.96, 27.7, 3.71, and 0.26, respectively, all substantially higher than that of Ran^WT^ (Table 1). We also analyzed three other Ran mutants Ran^Q69L^ (which is unable to hydrolyze GTP), Ran^1–179^ (without the 37 C-terminal residues), and Ran^1–210^ (without the C-terminus DEDDDL residues), with Bias_GTP_ of 0.15, 102.9, and 0.31, respectively (Supplementary Fig. 2). When the log scale of Bias_GTP_ was plotted against the % GTP quantified by Q column, an excellent Pearson Correlation Coefficient (CC = 0.90) was observed (Fig. 1c), suggesting that higher percent of bound GTP by C-dis Ran proteins was due to their higher relative affinities towards binding GTP.

### The C-dis mutants were able to interact with effector proteins

**Fig. 2:**
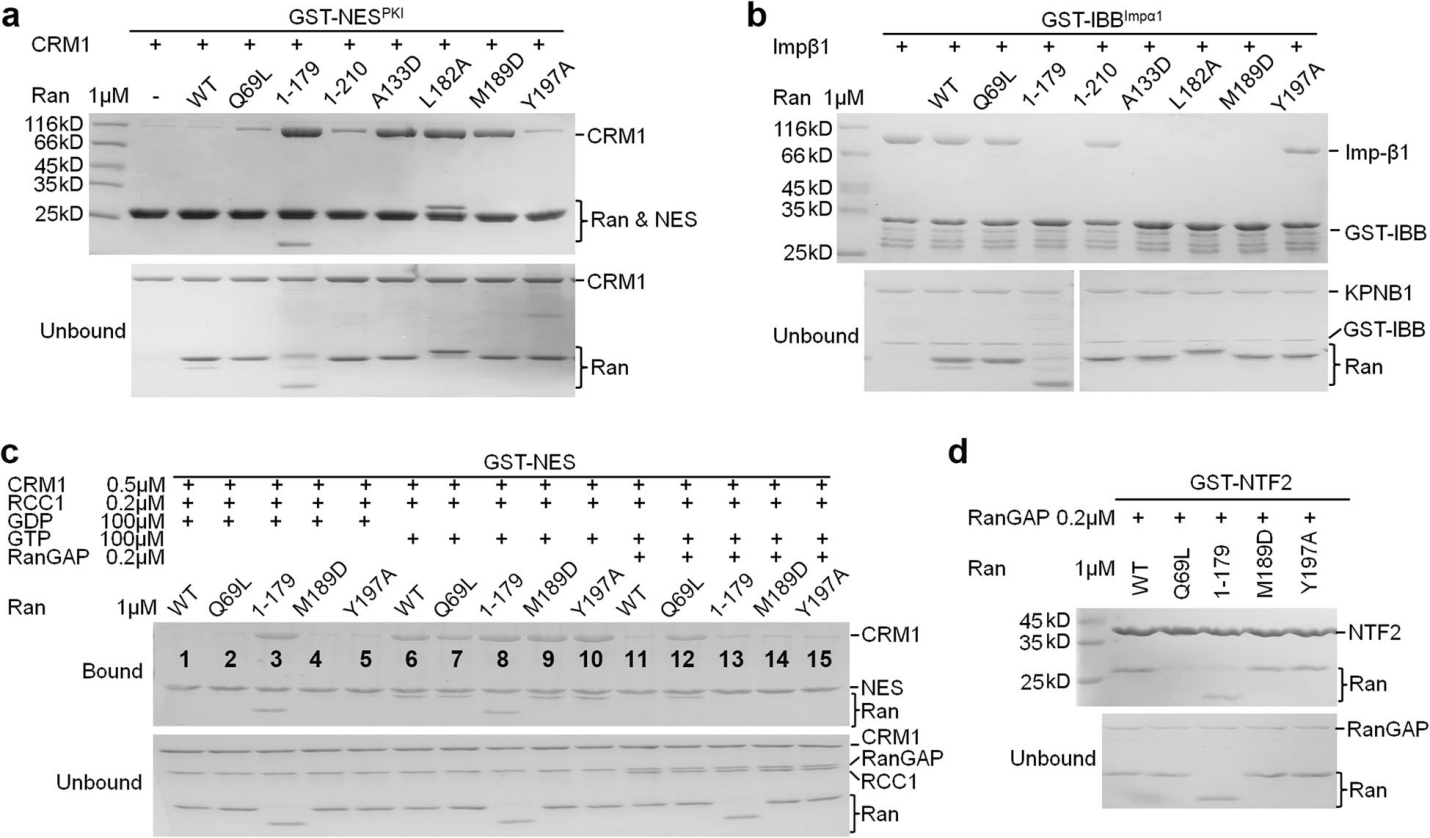
Comparison of different Ran mutants’ interaction with RCC1, RanGAP, and NTF2. **a** GST-NES^PKI^ pull-down of CRM1 and different Ran proteins. **b** GST-IBB^Impα1^ pull-down of Impβ1 in the presence of different Ran. RanGTP dissociates the Impβ1-IBB complex, so less Impβ1 means stronger Ran binding, in contrast to the CRM1 pull-down. **c** GST-NES^PKI^ pull-down of CRM1 and different Ran proteins, in the presence or absence of RCC1, RanGAP, GTP or GDP. **d** GST-NTF2 pull-down of different Ran proteins in the presence of RanGAP. RanGAP ensures all Ran proteins are in GDP-bound form (Purified Ran^Q69L^ is mainly GDP-charged)

RanGTP forms a nuclear export complex with CRM1 and NES cargo in the nucleus. Also in the nucleus, RanGTP dissociates importin β1 cargoes by directly binding to importin β1 (Impβ1). The ability of C-dis mutations to interact with CRM1-NES and Impβ1 was analyzed using pull-down. The C-dis mutants (A133D, L182A, and M189D) and Ran^1–179^ were much stronger in forming complexes with CRM1 or Impβ1, in agreement with their high level of bound GTP (Fig. 2a, b). Ran^Q69L^, Ran^1–210^, and Ran^Y197A^ mutants were slightly more potent in complex formation compared with Ran^WT^. The interaction of the C-dis mutant with other karyopherins is probably also retained, if not enhanced due to their higher GTP-binding level.

The GAP, GEF and nuclear import factor for Ran are RanGAP, RCC1, and NTF2, respectively. To know whether the mutant Ran proteins interact with RanGAP/RCC1/NTF2, we tested five representative proteins: Ran^WT^, Ran^Q69L^, Ran^1–179^, Ran^M189D^, and Ran^Y197A^. When incubated with either RCC1/GDP or RCC1/GTP, clear differences in the amount of bound CRM1 were observed for all Ran proteins (Fig. 2c, comparing lane 1–5 with 6–10), suggesting that these proteins responded to RCC1. Ran^1–179^ responded to RCC1 to a lesser extent (Supplementary Fig. 3). As expected, all Ran proteins were sensitive to the addition of RanGAP except Ran^Q69L^, which lacks the catalytic Q69 residue (Fig. 2c, comparing lane 6–10 with 11–15). When these Ran proteins were in the GDP-bound form (by the addition of RanGAP), all bound to NTF2 except Ran^Q69L^(Fig. 2d). Q69 was reported to be a critical NTF2 interacting residue [18]. In summary, C-dis mutants Ran^M189D^ and Ran^Y197A^ were able to be deactivated by GAP-mediated GTP hydrolysis, to respond to RCC1-mediated nucleotide exchange, and to bind to NTF2 when in the GDP-bound form.

### The C-dis mutants were more charged with GTP and more cytoplasmic in human cells

**Fig. 3:**
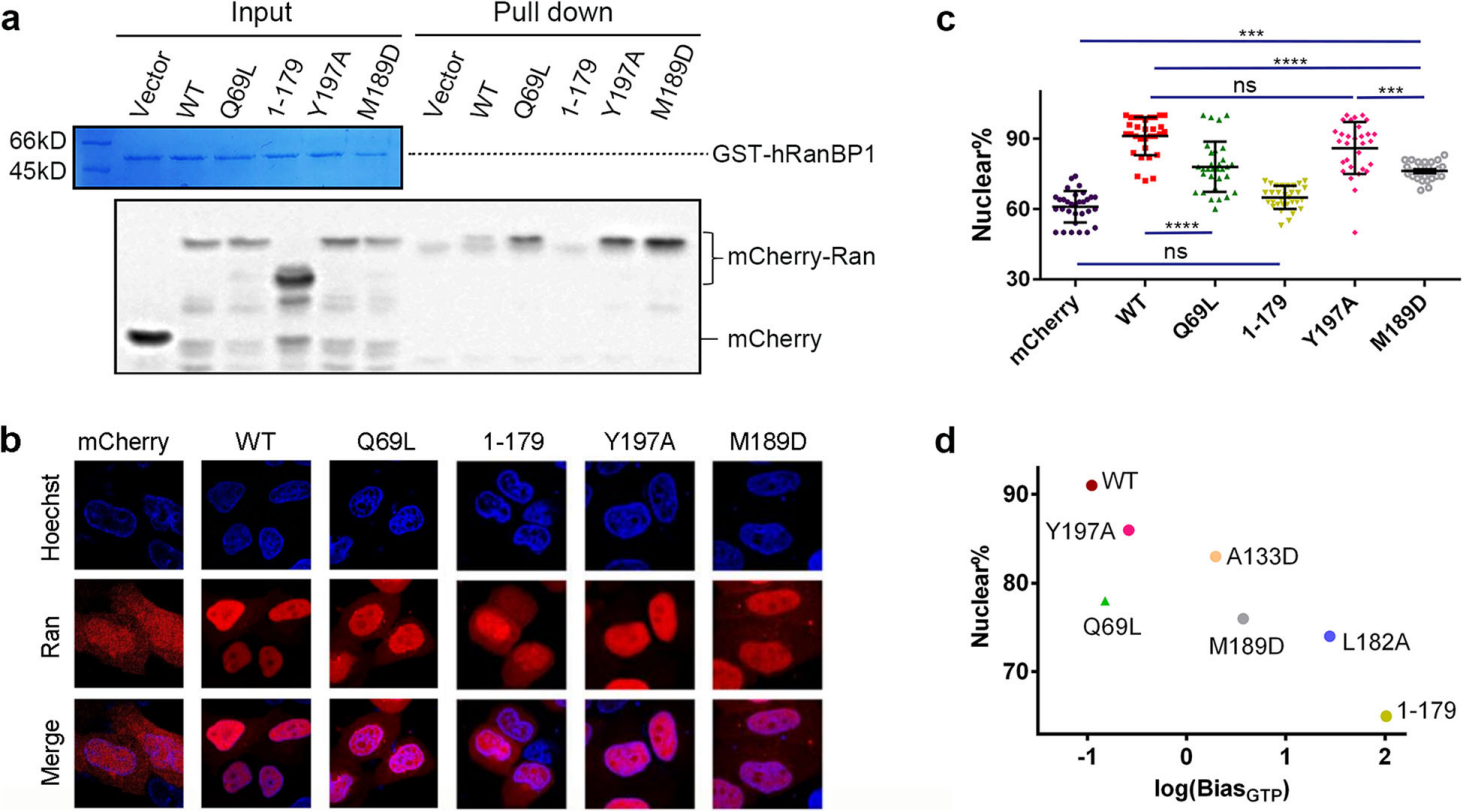
Greater GTP-loading level and cytoplasmic re-localization of Ran C-dis mutants in human cells. **a** GST-hRanBP1 pull-down of mCherry-Ran constructs transiently expressed in 293 T cells. Ran proteins were stained with mCherry antibody. Ran^1–179^ does not bind to RanBP1 because of lacking the C-terminus. The weak bands in Vector and 1–179 samples represent non-specific staining. **b** Intracellular localization of mCherry-tagged Ran proteins in transfected HeLa cells. **c** Quantification and statistical analysis of localization in panel (**b**). Percentage of nuclear Ran for each cell is calculated as Ran nuclear intensity divided by total cellular intensity. Middle horizontal lines represent the mean, and vertical lines represent the standard deviation of each set of data containing measurements from at least 30 cells. Levels of significance are analyzed by One-way ANOVA. **** *p <* 0.0001, *** *p* < 0.001, ** *p* < 0.01, * *p* < 0.05. **d** The negative correlation between the log scale of Bias_GTP_ and the percentage of nuclear Ran. The Pearson correlation coefficient for the seven proteins is − 0.86

To determine whether the effects on GTP-loading of the C-dis Ran mutants observed in vitro also occur in human cellular environments, we transfected 293 T cells with plasmids encoding N-terminal mCherry-tagged Ran proteins, lysed the cells, incubated the cleared whole cell lysate with immobilized GST-RanBP1, and blotted Ran using mCherry antibody (Fig. 3a). Compared with Ran^WT^, binding to RanBP1 was substantially increased for Ran^Q69L^, Ran^Y197A^, and Ran^M189D^, suggesting greater portion of those mutants were GTP-bound in cells. The binding of Ran^M189D^ to RanBP1 was much more than Ran^Q69L^, probably due to higher loading with GTP in cells.

Using mCherry-Ran transfection in HeLa cells, the subcellular localizations of these Ran proteins were analyzed. Compared to Ran^WT^ (91% nuclear), more Ran^Y197A^, Ran^Q69L^, Ran^M189D^, and Ran^1–179^ were localized to the cytoplasm (86%, 78%, 76% and 65% nuclear, respectively, Fig. 3b, c). Ran^M189D^ was significantly different from Ran^Y197A^ and mCherry (61% nuclear). Additionally, weak nuclear rim staining (because of hydrolysis incompetency) was observed for Ran^Q69L^ as previously reported [19], but not for the rest of the samples (which are hydrolysis-competent as shown in Fig. 2c). The localization of Ran^A133D^ and Ran^L182A^ in HeLa cells was further analyzed, which showed 83% and 74% nuclear, respectively (Supplementary Fig. 4). A negative correlation (CC = -0.86) was observed between Bias_GTP_ and the percentage of nuclear Ran (Fig. 3d), suggesting that the increased GTP-binding preference may be responsible for the phenotype of partial cytoplasmic localization.

### The C-dis mutants supported nuclear transport with slightly reduced efficiency

**Fig. 4:**
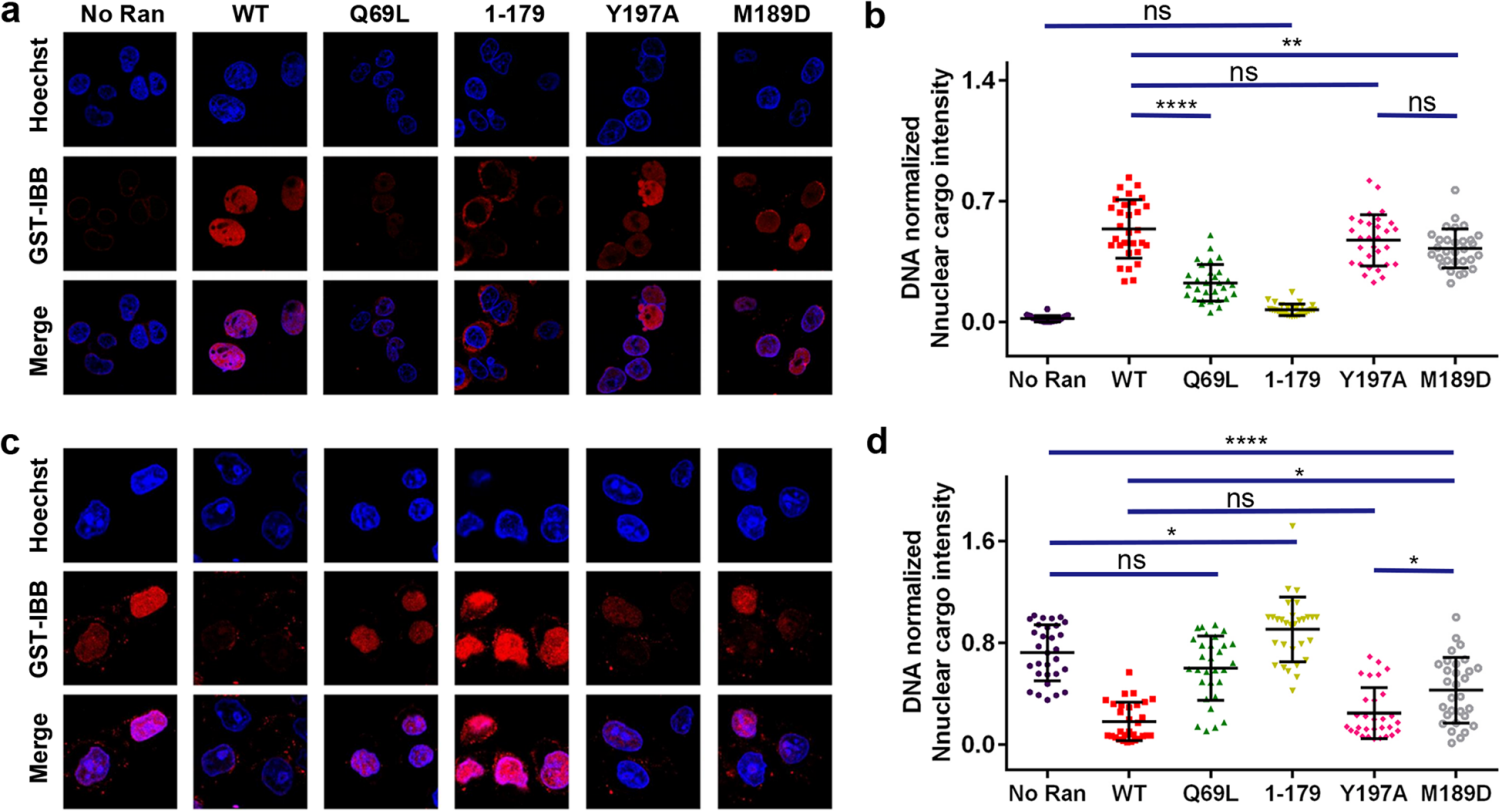
Slightly reduced efficiency in nuclear import and export by C-dis mutants in semi-permeabilized HeLa cells. **a** HeLa cells after nuclear import of GST-cargoes in the presence of Ran or different mutants were stained with anti-GST antibody (red). The GST-tagged importin β1 binding domain of importin α1 (GST-IBB) was used as the nuclear import cargo. For unknown reason, Ran^1–179^ is more prone to cytoplasmic staining, which theoretically should not be seen in semi-permeabilized cells. **b** Quantification and statistical analysis of nuclear import shown in panel (**a**). The level of nuclear import is assessed by nuclear cargo intensity (normalized with DNA intensity). **** *p* < 0.0001; ** *p* < 0.01; * *p* < 0.05. **c** HeLa cells after nuclear export of GST-cargoes in the presence of Ran and different mutants were stained with anti-GST antibody (red). GST-tagged human RanBP1 (GST-hRanBP1, which contains an NES) were used as the nuclear export cargo. **d** Quantification and statistical analysis of nuclear import shown in panel (**c**)

Using purified proteins and semi-permeabilized cells, the ability of these Ran proteins to facilitate nuclear transport of cargoes were assessed. Compared with ‘no Ran’ sample (mean:0.02), nuclear import of GST-IBB (Importin β1 Binding domain of importin α1) in the presence of importin β1 was stimulated by Ran^WT^ (0.53), partially by Ran^Q69L^ (0.22), but not by Ran^1–179^ (0.07) (Fig. 4a). Ran^Y197A^ (0.47) and Ran^M189D^ (0.42) were insignificant and significant (*p* < 0.01) compared with Ran^WT^, respectively (Fig. 4b). Between Ran^Y197A^ and Ran^M189D^, the level of nuclear import was statistically insignificant. For nuclear export of GST-hRanBP1 (which contains a NES) in the presence of nuclear export factor CRM1, only Ran^WT^, Ran^Y197A^, and Ran^M189D^ stimulated nuclear export (Fig. 4c). The difference in export activity relative to Ran^WT^ (0.18, the lower the number, the higher the export activity) was not significant with Ran^Y197A^ (0.24), but was significant with Ran^M189D^ (0.42, *p* < 0.05) (Fig. 4d). Ran^Y197A^ and Ran^M189D^ were significantly different from each other (*p* < 0.05). While Ran^Q69L^ (0.60) was not significantly different compared to the ‘no Ran’ sample (0.72), Ran^1–179^ (0.90, *p* < 0.05) showed further reduced nuclear export. In summary, the C-dis mutants function in nuclear transport with minimally or partially reduced efficiency.

### Several mild C-dis Ran cancer mutations were discovered

**Fig. 5:**
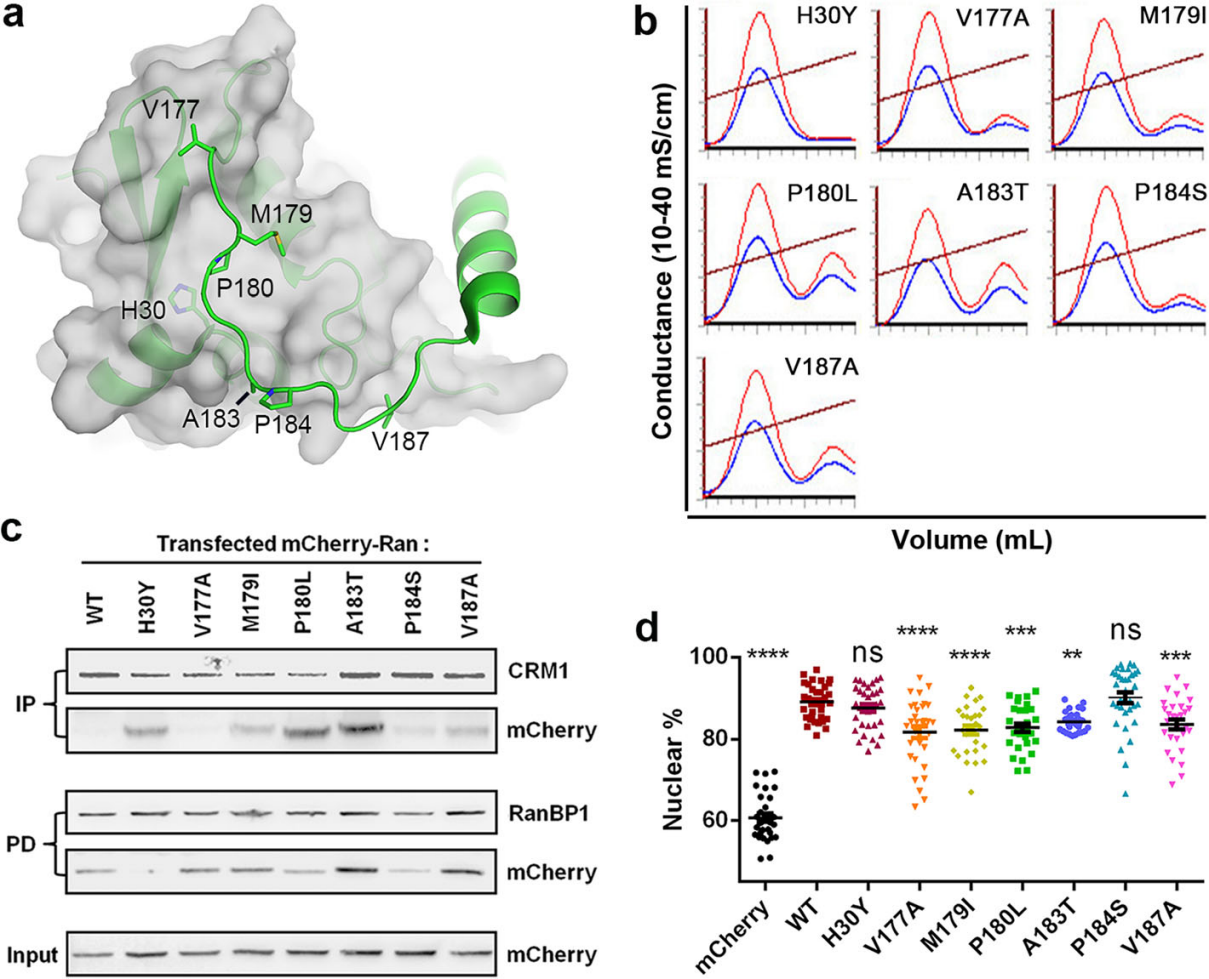
Discovery of multiple C-dis Ran mutants in cancers. **a** Location of mutated residues (stick) in cancers on the RanGDP crystal structure (3GJ0). Ran is displayed as a green cartoon with G-domain covered in partially transparent grey surface. **b** GTP% quantification by Q analysis for freshly purified cancer mutants. The brown line represents conductance. A260 and A280 are shown as red and blue curve, respectively. The peaks on the left and right represent GDP and GTP peaks, respectively. **c** CRM1 immunoprecipitation and GST-RanBP1 pull-down of transfected mCherry-Ran mutants in 293 T cells. **d** Subcellular localization of Ran cancer mutants. Error bars represents standard error of measurement (SEM). The statistical significance with Ran^WT^ sample is indicated at the top

Since Ran activation was previously shown to play a role in cellular transformation, the effects of Ran C-terminus cancer mutants were investigated. All mutations within residue range 177–187 in COSMIC and cBioPortal databases as well as H30Y were selected, since these residues might mediate the interaction between the C-tail and the G domain (Fig. 5a). These mutations were from various solid tumors, including kidney cancer, liver cancer, colon cancer, endometrial cancer, and skin cancer. Of these mutations, V177, M179, P180, A183, and V187 form hydrophobic interactions with the G-domain when Ran is GDP-bound. Core residue H30 is in direct contact with P180 in RanGDP. P184 is exposed to the solvent, but it may restrict the orientation/flexibility of the linker between C-helix and G domain. Our recent database search showed that P180L and V187A are no longer in the database.

The above discussed mutants were cloned and purified from *E. coli*. The percentage of bound GTP was assessed using Q anion exchange column. All mutants except Ran^H30Y^ showed a moderately (9–26%) increased level of bound GTP compared with Ran^WT^ (Fig. 5b, Supplementary Table 1). To minimize the influence of intrinsic hydrolysis before the protein was purified, we further engineered those mutations on top of the Ran^Q69L^ mutant and purified those double mutants together with Ran^Q69L^ from *E. coli*. All of the double mutants except Ran^Q69L/H30Y^ increased the level of bound GTP (7–68%) compared with Q69L single mutant (Supplementary Fig. 5). Though the level of bound GTP for each double mutant was generally increased compared with the respective single mutants, the correlation between the single and double mutant GTP% values was poor (CC = 0.45; see discussion). Further, the Bias_GTP_ values of the single mutants were analyzed as described earlier. The single mutants showed Bias_GTP_ values ranging from 0.20–0.55, all higher than Ran^WT^ (Table 1, Supplementary Fig. 6). In summary, most of the cancer mutants were slightly more biased to bind GTP compared with the WT protein.

### Several C-dis mutants were slightly activated and more cytoplasmic in human cells

We next transfected each mutant (fused with N-terminal mCherry) into 293 T cells and tested their interactions with CRM1 and RanBP1. These mutations did not cause obvious changes in expression level as judged by the input lanes. Except for V177A and P184S, more Ran mutants were immunoprecipitated relative to Ran^WT^, suggesting activation of those mutants (Fig. 5c). RanBP1 pull-down showed that the binding to RanBP1 was slightly increased for transfected A183T and V187A but reduced for H30Y and P184S. Thus, more cancer mutations showed enhanced binding to CRM1 than to RanBP1. The likely reason is that while all the mutation sites are distant from CRM1 interface (and thus do not perturb CRM1 affinity when in the GTP-bound form), residues H30, V177, P180, and P184 are in close contact with RanBP1 (and their mutations might thus reduce Ran’s binding to it). For example, the crystal structure of Ran-RanBP1 suggests that H30Y and P184S would not bind well to RanBP1 (Supplementary Fig. 7). The reduced RanBP1 binding strength (H30Y and P184S) may also account for Ran activation in cells, since binding to RanBP1 is required prior to RanGTP hydrolysis on the cytoplasmic side of NPC [2].

Cellular localization data showed that except for H30Y and P184S, the other cancer mutants were more localized to the cytoplasm compared to WT (Fig. 5d, Supplementary Fig. 8, 82–84% nuclear). Excluding the RanBP1 pull-down results, M179I, P180L, A183T and V187A were consistently activated as judged by GTP% values, Bias_GTP_, CRM1 immunoprecipitation and cellular localization.

### C-dis mutants Ran^A183T^ and Ran^V187A^ enhanced cell proliferation

**Fig. 6:**
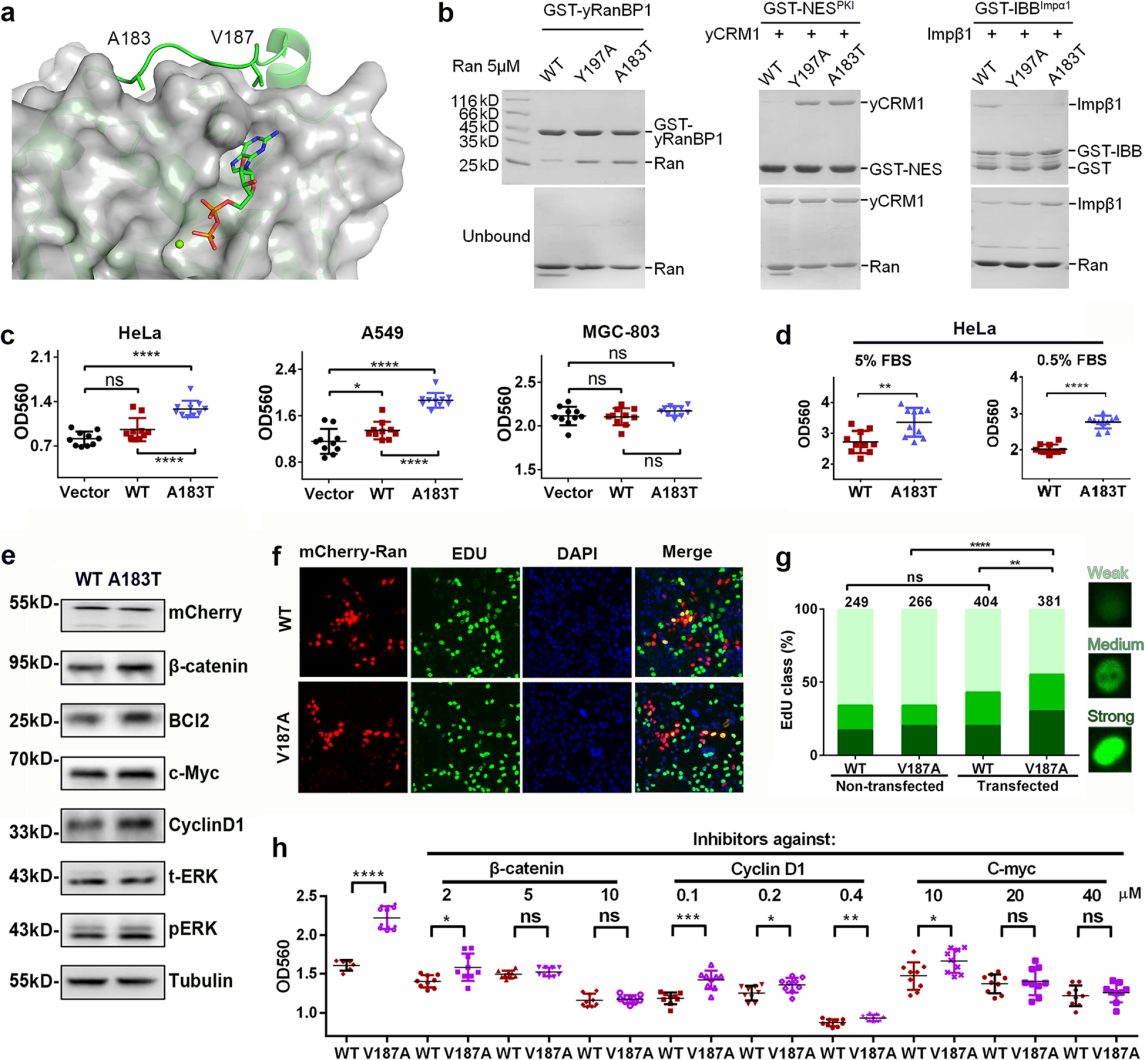
Enhanced cell proliferation by Ran^A183T^ and Ran^V187A^. **a** The interaction between the G domain and two C-terminal residues. **b** Comparing freshly purified Ran^WT^, Ran^A183T^ with Ran^Y197A^ binding activity by pull-down. Left: GST-yRanBP1 (yeast RanBP1) pull-down of Ran^WT^, Ran^Y197A^, and Ran^A183T^. Middle and right: GST-NES^PKI^ or GST-IBB^Impα1^ pull-down of yCRM1 or Impβ1 in the presence of Ran^WT^, Ran^Y197A^ and Ran^A183T^. **c** OD of transiently transfected (~ 50% efficiency) HeLa, A549, and MGC-803 cells respectively using SRB staining. Each transfection was performed with 12 replicates. **d** The normalized cell density of HeLa stable cell lines expressing mCherry-tagged Ran^WT^ or Ran^A183T^ in the presence of 0.5% or 5% FBS (fetal bovine serum). Y-axis shows the cell density at 72 h normalized by its density at 12 h. **e** Western analysis of mCherry-Ran stable cell lines (HeLa) grown at 0.5% FBS. **f** EdU staining of mCherry-WT or mCherry-Ran^V187A^ transiently transfected Hela cells. After three days of transfection, 10 μM EdU was added in the medium and incubated for 2 h. **g** Statistical analysis of the EdU staining results. ‘Non-transfected’ means the cells that were not showing mCherry signals in the wells of WT or V187A transfection. The numbers shown on top of the bar represent the total number of cells analyzed for each group. Statistical significance between groups was calculated by treating strong, medium, and weak EdU cells as 1, 2, 3, respectively. **h** The cell density of mCherry-WT or mCherry-V187A transfected HeLa in the presence of different inhibitors. Concentrations of 10,058-F4, Fascaplysin, and Nitazoxanide are shown in the plot

We next focused on two representative mutations A183T and V187A with slightly higher (0.40) and lower (0.22) Bias_GTP_ values, respectively. These two residues directly contact the G domain (Fig. 6a). Though mild activation is difficult to discern in a pull-down experiment, clear differences between Ran^A183T^ and Ran^WT^ were observed when higher concentrations of Ran proteins were used (Fig. 6b). Ran^A183T^ displayed a greater level of binding to RanBP1, CRM1 and Impβ1, similar to the engineered mutant Ran^Y197A^. We further investigated whether Ran^A183T^ could promote cellular transformation similar to the other reported Ran-activating mutations [13, 20]. Indeed, HeLa and A549 cells transfected with Ran^A183T^ displayed a higher growth rate than cells transfected with Ran^WT^ or vectors (Fig. 6c). Ran^WT^ transfection slightly enhanced the growth of A549 cells but not HeLa cells. In contrast, none of the transfections enhanced cell proliferation in MGC-803 cells, suggesting that the enhanced proliferation may be cell-type specific. In addition, mCherry-Ran^A183T^ transfected HeLa and A549 cells formed slightly more colonies than mCherry transfected cells (Supplementary Fig. 9).

To investigate the underlying mechanism, we next analyzed several proteins that were reported to be perturbed by Ran overexpression or activation, including Bcl-2 [21], ERK [12, 20], Cyclin D1 [22, 23], c-Myc [24] and β-catenin [25]. In both HeLa and A549 cells, Ran^A183T^ mildly increased the protein levels of β-catenin, c-Myc, Cyclin D1, and p-ERK (Supplementary Fig. 10). None of the analyzed proteins were substantially upregulated in Ran^A183T^-transfected MGC-803 cells. Overall, the western analysis agreed well with the cell proliferation data, suggesting that Ran^A183T^ promoted cell proliferation in HeLa and A549 cells, possibly through one or more of the pathways analyzed above.

We further generated HeLa stable cell lines expressing mCherry-Ran^WT^ or mCherry-Ran^A183T^. A higher cell proliferation rate for the Ran^A183T^ stable cell line was observed, which was potentiated with a low serum condition (Fig. 6d). Western analysis of the stable cell lines grown in the low serum condition showed similar results as transient transfection results, with mild upregulation of β-catenin, c-Myc, Cyclin D1, p-ERK, and Bcl-2 in A183T-expressing cells (Fig. 6e and Supplementary Fig. 11). The low serum grown cells were then treated with small molecule inhibitors against β-catenin, c-Myc, Cyclin D1, and p-ERK. Compared with Ran^WT^, treatment with β-catenin or Cyclin D1 inhibitors completely abolished the Ran^A183T^-induced cell proliferation advantage, while ERK or c-Myc inhibitors barely exerted any effect even at high concentrations (Supplementary Fig. 12).

To test whether a very small increase in Bias_GTP_ (such as V187A) plays a role in cell proliferation, an EdU incorporation experiment was performed with HeLa cells transiently transfected with mCherry-WT or mCherry-V187A (Fig. 6f). The results showed that V187A-transfected cells were slightly more often EdU positive. In the same well, V187A-transfected cells more often had strong EdU signals (30%) than non-transfected cells (20%, *p <* 0.0001) (Fig. 6g). In contrast, transfection with Ran^WT^ did not significantly increase the number of cells with strong EdU signals. HeLa cells transfected with mCherry-V187A also showed a higher cell proliferation rate than mCherry-WT-transfected cells, which could be dose-dependently diminished by the β-catenin or c-Myc inhibitor, but only partially by the Cyclin D1 inhibitor (Fig. 6h). These results suggest that even a slight increase in Bias_GTP_ may promote cell proliferation.

## Discussion

It turned out that the C-tail of Ran was very sensitive to mutation since all the designed mutations (A133D, L182A, M189D, and Y197A) increased Ran’s GTP-binding preference. The C-tail stabilizes Switch I in a GDP-favoring conformation [26]. The distances from L182, M189, Y197, A133, and the DEDDDL motif to Switch I are gradually increasing. The results indicate that closer residues were generally more important for GDP stabilization since Bias_GTP_ values trended to decrease for mutations at increasing distances. The most critical residue L182, which makes direct contact with Switch I, was responsible for a more than 200-fold GDP stabilization (0.11 vs. 27.7). The GDP-stabilization effect of each residue probably also depends on its G-domain binding energy including the contact area and the quality of the contact. The residues mutated by cancers contacted the G-domain much more weakly than those mutated by us, thus mutations of the former were generally less C-tail disrupting. Further, the outcomes of mutations at one site probably also depend on the type of mutation. For example, A133D mutation would probably show a greater Bias_GTP_ than A133G mutation since the former but not the latter would directly clash with the C-terminal helix. Overall, our results suggest that C-terminal residues contribute to autoinhibition to different degrees, and that the impact of mutations on these residues depends on multiple factors.

The Bias_GTP_ values agree well with the GTP% values purified from *E. coli* for the designed mutants. For cancer mutants, the correlation between Bias_GTP_ and GTP% was weaker (CC = 0.71), most notably for the H30Y mutant. The weaker correlation in cancer mutants was due in part to smaller signals, which is the changes relative to Ran^WT^. On the other hand, the designed mutants showed larger differences, yielding a higher signal-to-noise ratio assuming equal measurement errors. The Bias_GTP_ values are probably better indicators than GTP% values, mostly because of simpler assay condition. The concentrations of GTP and GDP fluctuate in *E. coli* [27]*,* which could influence the GTP% charged when Ran was expressed. There might be endogenous *E. coli* proteins that weakly and differently associated with different mutants, influencing their GTP/GDP relative affinity or GTP hydrolysis rate when Ran was expressed. These might explain the inconsistency of the GTP% values between the single mutants and the double mutants for the cancer mutations. Further, the Bias_GTP_ experiment is measured with a better precision (16 data points vs. 1 data point) and a broader range (0.11–103 vs. 0.06–0.87) than the GTP% experiment. To illustrate, 1–179, L182A, and M189D were barely distinguishable (87%, 85%, 84%) by the GTP% measurement, but showed a clear difference (102.9, 27.7, 3.71) by the Bias_GTP_ values.

Bias_GTP_’s correlation with the cellular data (cellular localization and nuclear export ability) were also weaker for both the engineered and the cancer mutants. Because the cellular environment is more complex, it is often seen that a clear in vitro phenomenon may only produce a weak and/or uneven response in cells. For examples, though Y197A showed obvious higher GTP binding preference in vitro, compared with Ran^WT^, its cellular localization and ability to support nuclear transport were not significantly different. Using the highly GTP-biased mutant such as M189D, it becomes clear that the C-dis mutants are more shifted to the cytoplasm and partially inhibit nuclear transport.

The C-tail of Ran is not involved in binding to RanGAP, RCC1, or NTF2 [18, 28, 29]. Therefore, the C-dis mutants were expected to interact with RanGAP/RCC1/NTF2 similarly to Ran^WT^. This agreed very well with our pull-down results. Contrary to the expectation that RanGTP predominates in the nucleus, the C-dis Ran mutants were observed to be more cytoplasmic compared than Ran^WT^. The cytoplasmic C-dis RanGTP could accumulate over a period of spontaneous nucleotide exchange in the GTP-rich cytoplasm [30]. In the cytoplasm, the higher GTP affinity increases the likelihood of loading a GTP at each spontaneous nucleotide exchange, eventually shifting the population to be more GTP-bound, just as in *E. coli* cells. Unless catalyzed by RanGAP which is localized only on the cytoplasmic side of NPC, cytoplasmic GTP-bound C-dis Ran would not bind to NTF2 for nuclear import and hence would remain in the cytoplasm.

Nuclear transport of proteins requires Ran to be able to switch on and off, and having a complete tail for interaction with RanBP1/RanBP2 facilitates cytoplasmic disassembly [31, 32]. In agreement, we showed that neither Ran^Q69L^ nor Ran^1–179^ was efficient in nuclear transport. Although the C-dis mutants were more likely to form complexes with karyopherins, they were actually less efficient in nuclear export. This is possibly due to the distortion of the nucleo-cytoplasmic RanGTP gradient (which is crucial for nuclear transport) and the reduced nuclear import efficiency of mutant Ran proteins (GTP-bound Ran would not be imported).

Instead of stimulating nuclear export, Ran^1–179^ inhibited nuclear export compared with buffer. One possible explanation is that Ran^1–179^ inhibits the speed of passive diffusion through nuclear pores, since Ran^1–179^ showed heavy nuclear rim staining in the semi-permeabilized cells. This nuclear rim staining suggests possible G domain interactions with stationary nups in the NPC under certain conditions. Ran^Q69L^ appeared as an outlier in the correlation plot between Bias_GTP_ and the nuclear ratio of Ran. This is because Ran^Q69L^ can not recognize its nuclear import factor NTF2 regardless of its nucleotide status [26], hence is more cytoplasmic. Q69L’s inability of NTF2 interaction and nuclear import would also partially account for the observed low efficiency in nuclear import/export, in addition to its hydrolysis incompetency.

Confidence in the assessment that cancer mutants are weakly activated could be strengthened by combing different techniques such as GTP% values, Bias_GTP_, CRM1 immunoprecipitation, and cellular localization. Albeit infrequent in cancers, most of the tested cancer mutants were more biased to bind to GTP and more GTP-bound compared to Ran^WT^, suggesting that C-dis mutations do exist in nature. Interestingly, highly C-disrupting Ran cancer mutations like M189D were not discovered among the tested mutants. We hypothesize that highly GTP-charged Ran mutants may distort the RanGTP gradient and inhibit nuclear transport like M189D, thus retarding cell growth.

Ran is overexpressed in a few cancers [13, 15, 22, 23]. Further, downregulation of Ran inhibits cell proliferation, invasion and metastasis [22, 33]. Here we showed that overexpression of Ran^WT^ could itself slightly promote cell proliferation in A549 cells. The overexpression of C-dis mutant A183T could enhance cell proliferation both in A549 and HeLa cells. In the Ran^A183T^-expressing HeLa stable cell line, the stimulated cell proliferation is more apparent in low serum growth conditions, similar to a previously reported GTP-destabilizing Ran mutant [20]. Although the cells lines (HeLa, A549) used were not the adenocarcinoma cells in which A183T was observed, our results served as a proof of concept that C-dis mutations can promote cell proliferation in vitro. Further studies in adenocarcinoma cells may ascertain the in vivo function of Ran C-dis activation.

Upregulation of nuclear transport (import and export) often plays important roles in cancers [34]. Activation of Ran, however, was shown to slightly inhibit both nuclear export and nuclear import as shown and discussed earlier. Therefore, it is unlikely that the activation of Ran stimulated cell growth through upregulation of nuclear transport. Since cancer mutations shifted a fraction of nuclear RanGTP to the cytoplasm, the enhanced cell proliferation might be mediated by Ran’s cytoplasmic functions [35]. Our pilot work with inhibitors against upregulated proteins showed that elevated β-catenin levels might be responsible for the enhanced HeLa cell proliferation by both A183T and V187A. It may be worth validating and clarifying the full path of Ran C-dis activation in cancers.

It should be noted that the observed cellular activation of C-dis Ran is independent of GEF/GAP, which is considered to be the primary regulation mechanism of small GTPase. For the first time, it is shown that Ran contains another level of cellular regulation through impairment of C-terminal autoinhibition. In human cells, post-translational modifications (PTMs) may exist that regulate the strength of C-tail autoinhibition and the GTP preference of Ran, leading to its activation/inactivation. Though Ran is the only GTPase that has an autoinhibitory C-terminus, other GTPases may also harbor mutations that perturb their GTP/GDP preference. Such mutations might influence the cellular activation level of a GTPase, its interaction with the signaling cascades, and finally cell fate. Whether such mutations exist in other small GTPases warrants further studies.

## Materials and methods

### Mant-GDP dissociation assay

Different fresh proteins (2.5 μM, loaded with varying ratios of GTP or GDP, approximately 10% apo) were first incubated with 2.5 μM of mant-GDP and 0.05 μM of RCC1 at room temperature for 20 min so that the proteins were theoretically charged with more than 50% of mant-GDP on 96 well plates. Ran mutants which were more biased to bind GTP were charged with mant-GTP instead of mant-GDP. The fluorescence intensity of mant-GDP increases upon association with Ran. Increasing concentrations of GDP or GTP (5.0 μM to 1.25 mM, 7 concentrations, 20 μL each) were added to 80 μL protein in each well, incubated for 30 min at room temperature and read using a fluorescent microplate reader (BioTek). Each concentration was run in triplicate. The assay was conducted in buffer containing 40 mM HEPES pH 7.5, 200 mM NaCl, and 5 mM MgCl_2_.

The titrations were normalized pairwise (GDP/GTP). The average of the smallest readings (which are the highest GDP concentration readings for GDP binder, and vice versa) was first subtracted for all readings. The resulting values were normalized by the average of buffer sample readings (no mant-GDP dissociation). To avoid ambiguities, the data were then converted by subtracting each normalized value from 100%, then fitted using four-parameter logistic regression in GraphPad software. Error bars represent standard deviations of triplicates.

### Immuno-precipitation and confocal microscopy

Cells were maintained in Dulbecco’s modified Eagles medium (Hyclone) supplemented with 10% (v/v) fetal bovine serum (Biological Industries) and transfected with TurboFect transfection reagent (Thermo Scientific). CRM1 (ProteinTech) and mCherry (ProteinTech) antibodies were used at 1:5000 and 1:1000 dilutions, respectively. Images were acquired with an Olympus FV-1000 confocal microscope and were analyzed using NIH ImageJ and Graphpad software.

### Western blot and cell proliferation assay

HeLa, MGC803, A549 and 293 T cells were maintained in Dulbecco’s modified Eagles medium (Hyclone) supplemented with 10% (v/v) fetal bovine serum (Biological Industries) and transfected with polyethyleneimine (PEI) transfection reagent (Thermo Scientific). Cell proliferation assay was performed with 96-well plates, seeding about 1500 cells for each well. Every plasmid was transfected in 10–12 replicates. Sulforhodamine B (SRB) staining was performed 5 days after transfection. Western blot was performed with 6-well plates, 48 h after transfection. Antibodies against Tubulin (ProteinTech,1:5000), mCherry (ProteinTech, 1:1000), Bcl-2 (SAB, 1:1000), t-ERK (SAB, 1:1000), p-ERK (SAB, 1:1000), β-catenin (SAB, 1:1000), CyclinD1 (ProteinTech, 1:1000) and c-Myc (ProteinTech, 1:1000) were used. Stable cell lines were generated using lentiviral infection of HeLa cells in pLVX-IRES-Neo plasmids. Inhibitors against ERK (PD98059, Selleck), c-Myc (10058-F4, MCE), Cyclin D1 (Fascaplysin, Santa Cruz) and β-catenin (Nitazoxanide, Selleck) were purchased.

### In vitro nuclear transport using semi-permeabilized cells

The in vitro nuclear import assay was slightly modified from an earlier report [36]. Briefly, the cells were first semi-permeabilized with 50 ng/mL digitonin in 12-well plates. After washing off the soluble cell contents, 1 μM GST-IBB, 0.5 μM importin β1, 1 μM NTF2, 1× energy regeneration system [36], 0.01% Triton-X100 (to prevent non-specific binding), and 2 μM of different Ran proteins were added to semi-permeabilized HeLa cells and incubated at room temperature for 60 min. After the reaction, the cells were washed, fixed, and visualized by immunostaining with GST antibody. For the nuclear export assay, semi-permeabilized HeLa cells were first incubated with 1 μM GST-hRanBP1, 2 μM Ran^WT^, 1 × energy regeneration system, and 0.01% Triton-X100 for 60 min in order to accumulate nuclear GST-hRanBP1. The cells were then washed and incubated with 1 μM of hCRM1, 1× energy regeneration system, 0.01% Triton-X100, and 2 μM of different Ran proteins for 30 min at room temperature with gentle shaking. After the incubation, cells were washed, fixed, and visualized by immunostaining with a GST antibody. Statistics were based on measurements from at least 30 cells for each sample, and statistical significance was calculated by one-way ANOVA test in GraphPad software.

## Supplementary information

**Additional file 1: Supplementary Table 1.** Overview table of some results obtained in this study. **Supplementary Figure 1.** GST-IBB pull down of Impβ1 in the presence of Ran^WT^ (A) or Ran^M189D^ (B), pre-incubated with RCC1 and varying ratio of GDP/GTP (total 50 μM). Ran proteins were first incubated with small amount of RCC1 and different ratio of GTP and GDP, then the percentage of GTP-bound Ran was measured by testing whether it could disrupt the binding between GST-IBB^Impα1^ and Impβ1. At GTP:GDP ratio of 9.4, the concentration of activated Ran^WT^ exceeds that of Impβ1 (which is 20% of Ran concentration) and thus fully inhibited its binding to GST-IBB^Impα1^ (A). On the other hand, a small GTP:GDP ratio (0.21, calculated as 1/4.7) is able to charge more than 20% Ran^M189D^ with GTP (B). It is thus estimated that M189D mutation increased the relative affinity for GTP over GDP by at least ten folds. **Supplementary Figure 2.** mant-GDP dissociation by different concentrations of GDP or GTP. Error bars represent standard deviations of triplicates. IC50 values are shown in Table 1. **Supplementary Figure 3.** GST-NES pull down of yCRM1 and Ran^1–179^ in the presence of GDP and different concentrations of RCC1. yCRM1 and Ran^1–179^ were bound much less when the concentration of RCC1 is increased to 2 μM. **Supplementary Figure 4.** Cellular localization of transfected mCherry-A133D and mCherry-L182A in HeLa cells. The right panel shows the quantification of the corresponding nuclear ratio of localization (means: 0.83 and 0.74 respectively). The nuclear Ran ratio for each cell is calculated as Ran nuclear intensity divided by total cellular intensity. Middle horizontal lines represent the mean, and vertical lines represent the standard deviation of each set of data containing measurements from at least 28 cells. **Supplementary Figure 5.** GTP% quantification by Q anion exchange analysis. Except Q69L, the rest are double mutants based on Q69L. Most mutations showed increased level of bound GTP compared with the respective single mutants (Fig. 5b). **Supplementary Figure 6.** Profile of mant-GDP dissociation by different concentrations of GDP or GTP on naturally occurring cancer mutations. Error bars represent standard deviations of triplicates. **Supplementary Figure 7.** Cancer mutations P184S and H30Y might inhibit RanBP1 binding. Shown is the Ran-RanBP1 complex in pdb 1K5G. RanBP1 is shown as an electron static surface potential map. Ran is shown as cartoon representation. P184 and H30 are shown as sticks. **Supplementary Figure 8.** Cellular localization of cancer mutants in transfected HeLa cells. Ran was fused with an N-terminal mCherry fusion. The quantification and statistical analysis is shown in Fig. 5d. **Supplementary Figure 9.** Colony formed by mCherry-only or mCherry-WT or mCherry-A183T transfected HeLa and A549 cells. About 2000 HeLa or A549 cells were plated on 6-well plates. The plasmids were repeatedly (every two to three days) transfected into cells to ensure high transformation rate. Cells were stained using crystal violet 14 days after plating. **Supplementary Figure 10.** Protein levels by mCherry-only or mCherry-WT or mCherry-A183T transformation in HeLa, A549, and MGC-803 cells. **Supplementary Figure 11.** Quantification and statistical analysis of gel bands shown in Fig. 6e. The quantification is performed in triplicate, varying the size of integration box. Each group is normalized by the average of three WT intensities. *** *p* < 0.001; ** *p* < 0.01; * *p* < 0.05. **Supplementary Figure 12.** The normalized density of HeLa stable cells (WT or A183T) in the presence of 0.5% FBS and different inhibitors. Y-axis shows the cell density at 72 h normalized by its density at 12 h. PD98059 (50 μM), 10,058-F4 (20 μM), Fascaplysin (0.1 μM), and Nitazoxanide (5 μM) are inhibitors against ERK, c-Myc, Cyclin D1, and β-catenin, respectively.

## Data Availability

All data generated or analyzed during this study are included in this published article and its supplementary information files.

## Abbreviations

CC: Correlation coefficient
C-dis: C-terminal tail disrupting
CRM1: Chromosome region maintenance 1
EdU: 5-Ethynyl-2′-deoxyuridine
GAP: GTPase activating protein
GDP: Guanosine diphosphate
GEF: Guanine nucleotide exchange factor
GST: Glutathione S-transferase
GTP: Guanosine triphosphate
HEPES: 4-(2-Hydroxyethyl)-1-piperazineethanesulfonic acid
IBB: Importin β1 Binding domain of importin α1
NES: Nuclear export signal
NLS: Nuclear localization signal
NPC: Nuclear pore complex
NTF2: Nuclear transport factor 2
Ran: Ras-related nuclear protein
RCC1: Regulator of chromosome condensation
SRB: Sulforhodamine B
WT: Wild type
