# Supplementary material for: GEF-independent Ran activation shifts a fraction of the protein to the cytoplasm and promotes cell proliferation
Source: Mol Biomed. 2020 Dec 30;1:18. doi: 10.1186/s43556-020-00011-2 (PMC8607414; doi:10.1186/s43556-020-00011-2)
Supplement: Supplementary file 1 — Additional file 1: Supplementary Table 1. Overview table of some results obtained in this study. Supplementary Figure 1. GST-IBB pull down of Impβ1 in the presence of RanWT (A) or RanM189D (B), pre-incubated with RCC1 and varying ratio of GDP/GTP (total 50 μM). Ran proteins were first incubated with small amount of RCC1 and different ratio of GTP and GDP, then the percentage of GTP-bound Ran was measured by testing whether it could disrupt the binding between GST-IBBImpα1 and Impβ1. At GTP:GDP ratio of 9.4, the concentration of activated RanWT exceeds that of Impβ1 (which is 20% of Ran concentration) and thus fully inhibited its binding to GST-IBBImpα1 (A). On the other hand, a small GTP:GDP ratio (0.21, calculated as 1/4.7) is able to charge more than 20% RanM189D with GTP (B). It is thus estimated that M189D mutation increased the relative affinity for GTP over GDP by at least ten folds. Supplementary Figure 2. mant-GDP dissociation by different concentrations of GDP or GTP. Error bars represent standard deviations of triplicates. IC50 values are shown in Table 1. Supplementary Figure 3. GST-NES pull down of yCRM1 and Ran1–179 in the presence of GDP and different concentrations of RCC1. yCRM1 and Ran1–179 were bound much less when the concentration of RCC1 is increased to 2 μM. Supplementary Figure 4. Cellular localization of transfected mCherry-A133D and mCherry-L182A in HeLa cells. The right panel shows the quantification of the corresponding nuclear ratio of localization (means: 0.83 and 0.74 respectively). The nuclear Ran ratio for each cell is calculated as Ran nuclear intensity divided by total cellular intensity. Middle horizontal lines represent the mean, and vertical lines represent the standard deviation of each set of data containing measurements from at least 28 cells. Supplementary Figure 5. GTP% quantification by Q anion exchange analysis. Except Q69L, the rest are double mutants based on Q69L. Most mutations showed increased level of bound GTP compa [file 43556_2020_11_MOESM1_ESM.docx]

# Supplementary Information for

**GEF-independent Ran activation shifts a fraction of the protein to the cytoplasm and promotes cell proliferation**

Jinhan Zhou^1#^, Yuping Tan^1#^, Yuqing Zhang^1#^, Aiping Tong^1^, Xiaofei Shen^2^, Xiaodong Sun^3^, Da Jia^2*^, Qingxiang Sun^1^*

^1^Department of Pathology, State Key Laboratory of Biotherapy and Cancer Centre, West China Hospital, Sichuan University and Collaborative Innovation Center of Biotherapy, Chengdu 610041, China

^2^Key Laboratory of Birth Defects and Related Diseases of Women and Children, Department of Paediatrics, Division of Neurology, West China Second University Hospital, Sichuan University, Chengdu 610041, China

^3^ Department of Pharmacology, West China School of Basic Medical Sciences and Forensic Medicine, Sichuan University, Chengdu 610041, China

#Equal contribution

*Corresponding author: [qingxiang.sun@scu.edu.cn](mailto:qingxiang.sun@scu.edu.cn) ORCID 0000-0002-9474-8882

or [jiada@scu.edu.cn](mailto:jiada@scu.edu.cn) ORCID 0000-0002-2205-1998

This file contains:

Supplementary Table 1

Supplementary Fig. 1-12

Supplementary Table 1. Overview table of some results obtained in this study.

|  | Mutation | GTP%^1^ | Bias_GTP_ |  | Bound CRM1^3^ | Bound RanBP1^3^ | Nuclear%^4^ | Nuclear import^5^ | Nuclear export^5^ |
| --- | --- | --- | --- | --- | --- | --- | --- | --- | --- |
|  | WT | 6 | 0.11 |  | - | - | 91 | 0.54 | 0.18 |
| Reported Mutations | Q69L | 12 | 0.15 |  | + | + | 78 | 0.22 | 0.60 |
|  | 1-179 | 87 | 102.9 |  | +++ | - | 65 | 0.07 | 0.90 |
|  | 1-210 | 35 | 0.31 |  | + | - |  |  |  |
| Designed Mutations | A133D | 79 | 1.96 |  | +++ | +++ | 83 |  |  |
|  | L182A | 85 | 27.7 |  | +++ | +++ | 74 |  |  |
|  | M189D | 84 | 3.71 |  | +++ | +++ | 76 | 0.47 | 0.43 |
|  | Y197A | 23 | 0.26 |  | + | + | 86 | 0.42 | 0.25 |
| Cancer Mutations | H30Y | 0 | 0.20 |  | +++ | - | 88 |  |  |
|  | V177A | 17 | 0.25 |  | + | + | 82 |  |  |
|  | M179I | 15 | 0.20 |  | ++ | + | 83 |  |  |
|  | P180L | 32 | 0.55 |  | +++ | + | 83 |  |  |
|  | A183T | 23 | 0.40 |  | +++ | ++ | 84 |  |  |
|  | P184S | 15 | 0.20 |  | + | -/+ | 90 |  |  |
|  | V187A | 26 | 0.22 |  | ++ | ++ | 83 |  |  |

1. Calculated as GTP/(GDP+GTP). The GTP% values for WT, the reported, and the designed mutations were from one of our recent work.

2. Performed at 37 °C in the presence of GTP.

3. These are not the intrinsic property. For reported and designed mutations, +/- signs reflect the amount of bound GTP in freshly purified proteins. For cancer mutations, the signs reflect the amount of bound GTP in 293T expressed mCherry fusions (similar level of expression).

4. The proteins are fused with an N-terminal mCherry tag. mCherry itself is 61% nuclear.

5. Values are the mean nuclear cargo intensity (normalized by DNA intensity). Higher numbers for nuclear import means higher import level, and lower numbers for export means higher export level.


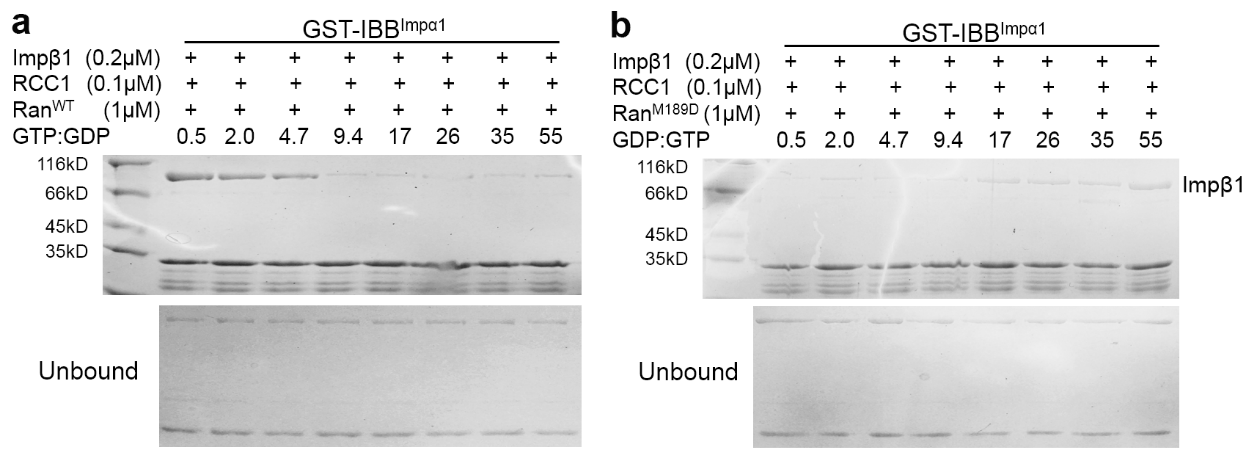


Supplementary Fig. 1 GST-IBB pull down of Impβ1 in the presence of Ran^WT^ (A) or Ran^M189D^ (B), pre-incubated with RCC1 and varying ratio of GDP/GTP (total 50 µM). Ran proteins were first incubated with small amount of RCC1 and different ratio of GTP and GDP, then the percentage of GTP-bound Ran was measured by testing whether it could disrupt the binding between GST-IBB^Impα1^ and Impβ1. At GTP:GDP ratio of 9.4, the concentration of activated Ran^WT^ exceeds that of Impβ1 (which is 20% of Ran concentration) and thus fully inhibited its binding to GST-IBB ^Impα1^ (A). On the other hand, a small GTP:GDP ratio (0.21, calculated as 1/4.7) is able to charge more than 20% Ran^M189D^ with GTP (B). It is thus estimated that M189D mutation increased the relative affinity for GTP over GDP by at least ten folds.


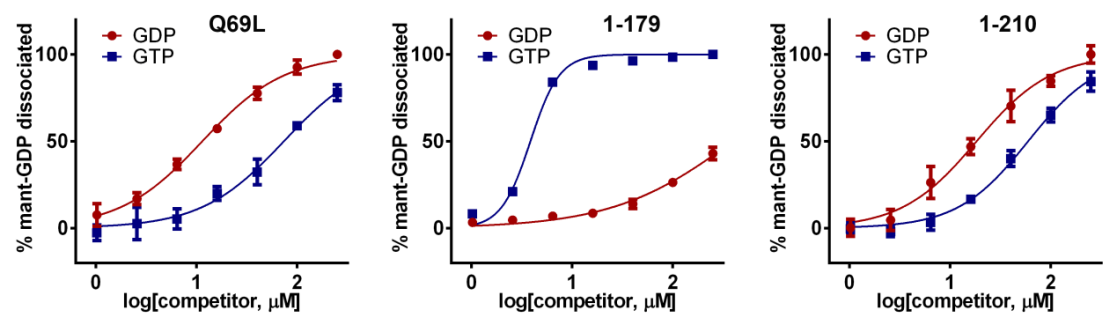


Supplementary Fig. 2 mant-GDP dissociation by different concentrations of GDP or GTP. Error bars represent standard deviations of triplicates. IC50 values are shown in Table 1.


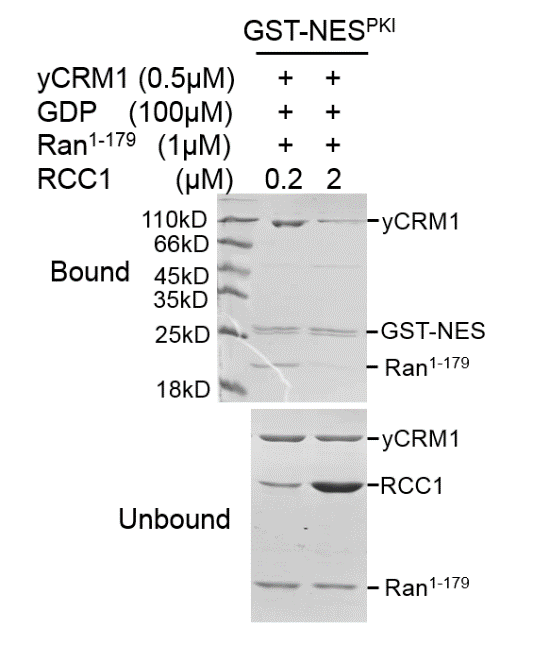


Supplementary Fig. 3 GST-NES pull down of yCRM1 and Ran^1-179^ in the presence of GDP and different concentrations of RCC1. yCRM1 and Ran^1-179^ were bound much less when the concentration of RCC1 is increased to 2 µM.


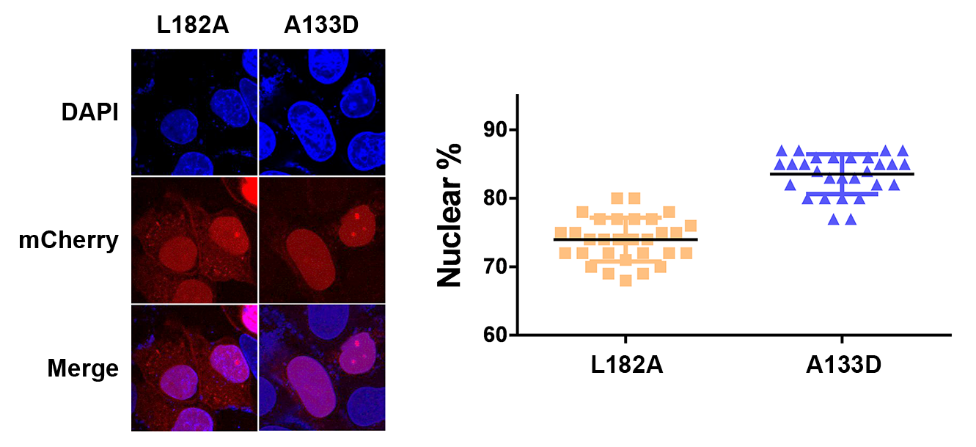


Supplementary Fig. 4 Cellular localization of transfected mCherry-A133D and mCherry-L182A in HeLa cells. The right panel shows the quantification of the corresponding nuclear ratio of localization (means: 0.83 and 0.74 respectively). The nuclear Ran ratio for each cell is calculated as Ran nuclear intensity divided by total cellular intensity. Middle horizontal lines represent the mean, and vertical lines represent the standard deviation of each set of data containing measurements from at least 28 cells.


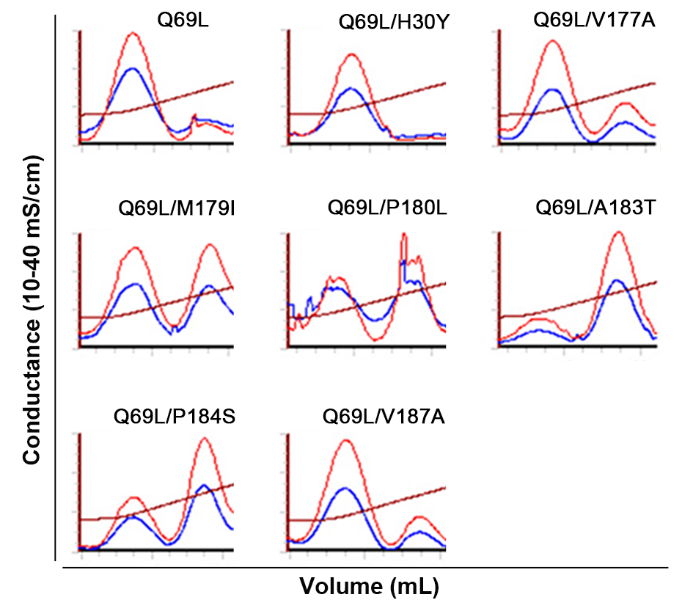


Supplementary Fig. 5 GTP% quantification by Q anion exchange analysis. Except Q69L, the rest are double mutants based on Q69L. Most mutations showed increased level of bound GTP compared with the respective single mutants (Fig. 5B).


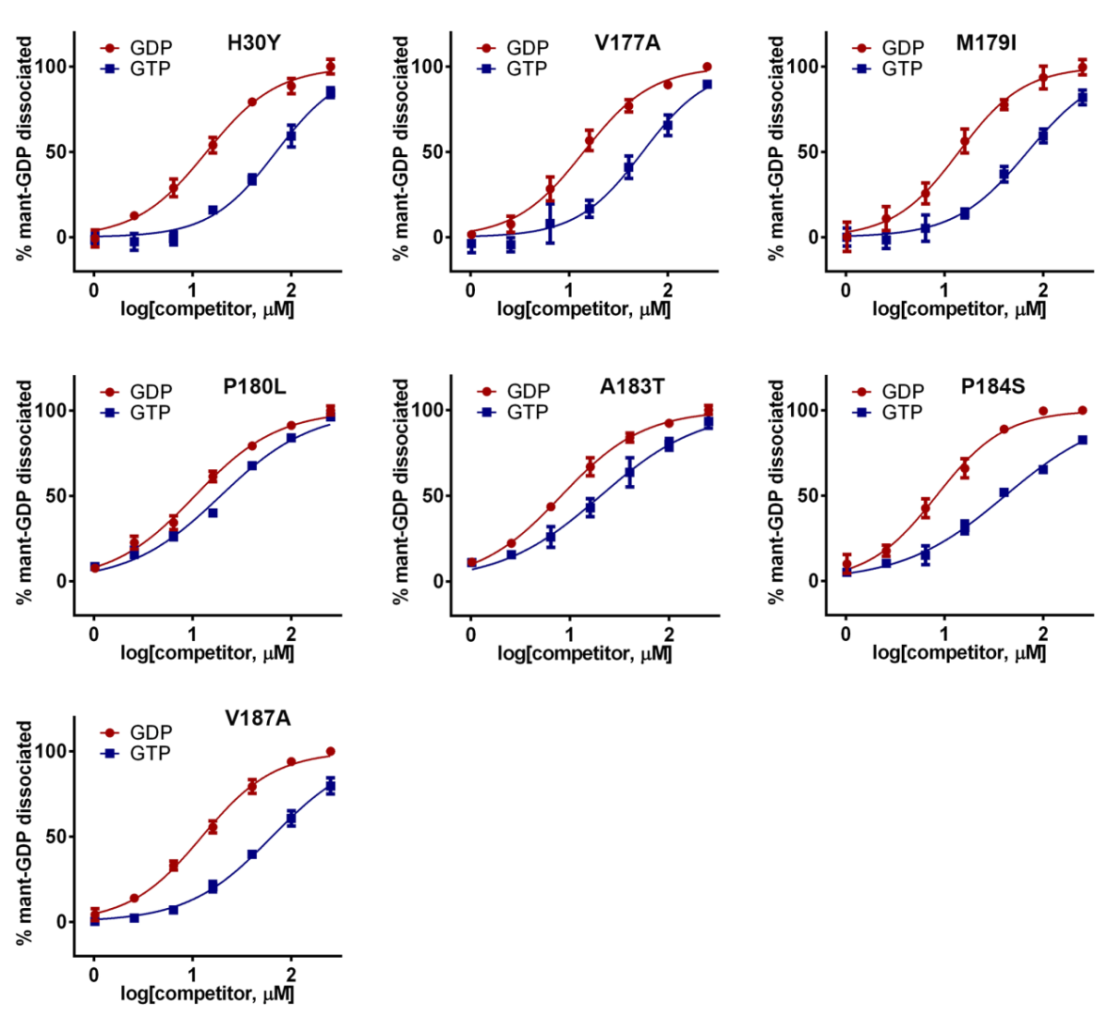


Supplementary Fig. 6 Profile of mant-GDP dissociation by different concentrations of GDP or GTP on naturally occurring cancer mutations. Error bars represent standard deviations of triplicates.


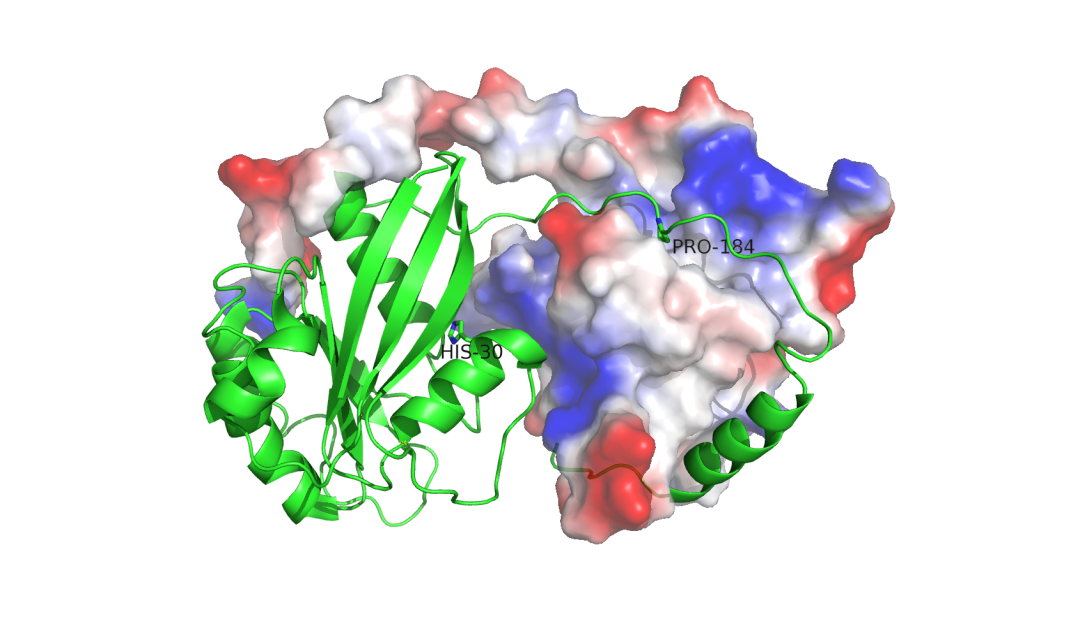


Supplementary Fig. 7 Cancer mutations P184S and H30Y might inhibit RanBP1 binding. Shown is the Ran-RanBP1 complex in pdb 1K5G. RanBP1 is shown as an electron static surface potential map. Ran is shown as cartoon representation. P184 and H30 are shown as sticks.


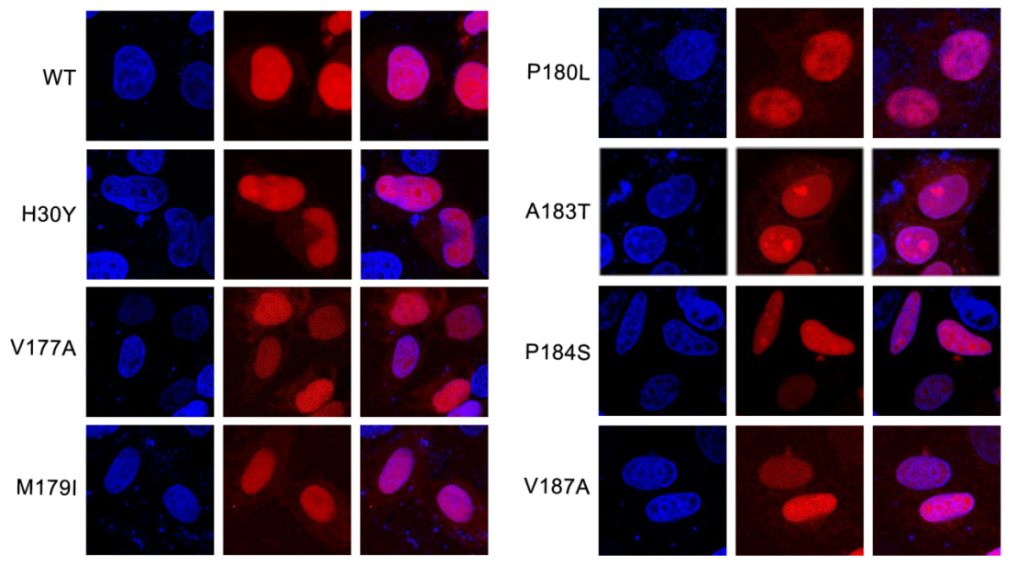


Supplementary Fig. 8 Cellular localization of cancer mutants in transfected HeLa cells. Ran was fused with an N-terminal mCherry fusion. The quantification and statistical analysis is shown in Fig. 5D.


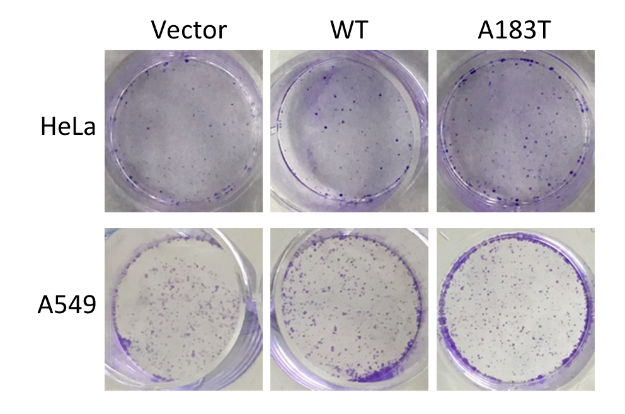


Supplementary Fig. 9 Colony formed by mCherry-only or mCherry-WT or mCherry-A183T transfected HeLa and A549 cells. About 2000 HeLa or A549 cells were plated on 6-well plates. The plasmids were repeatedly (every two to three days) transfected into cells to ensure high transformation rate. Cells were stained using crystal violet 14 days after plating.


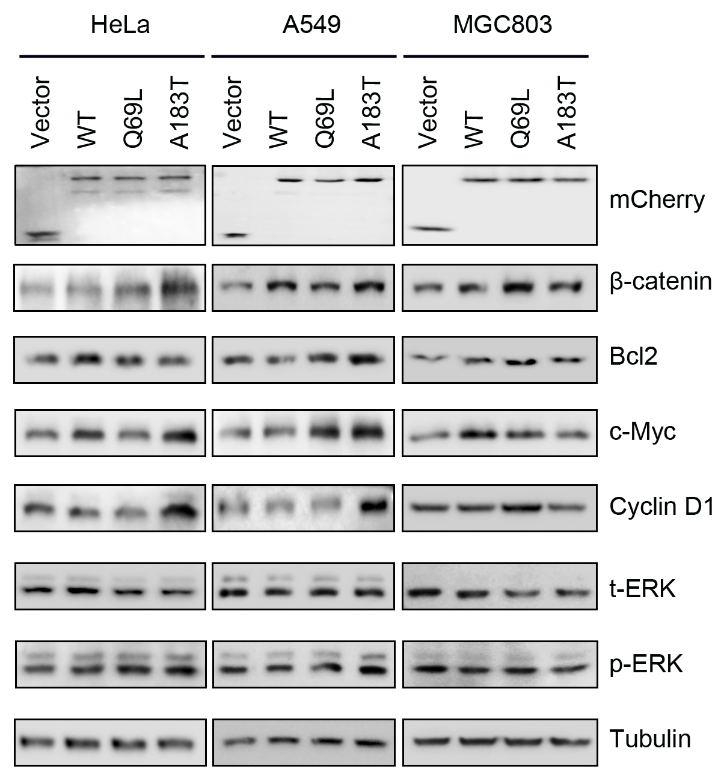


Supplementary Fig. 10 Protein levels by mCherry-only or mCherry-WT or mCherry-A183T transformation in HeLa, A549, and MGC-803 cells. This experiment was performed along with Ran^Q69L^, which is not relavent in this context.


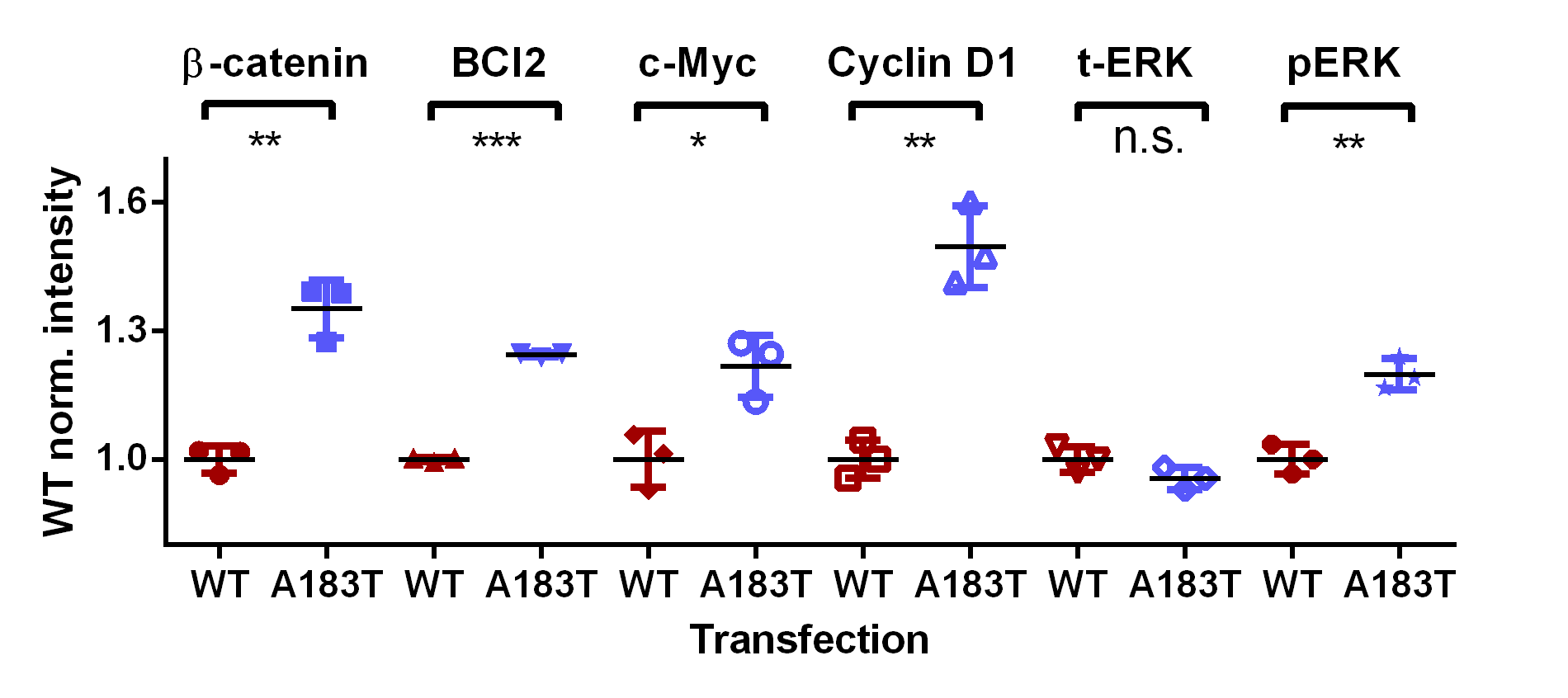


Supplementary Fig. 11 Quantification and statistical analysis of gel bands shown in Figure 6E. The quantification is performed in triplicate, varying the size of integration box. Each group is normalized by the average of three WT intensities. *** p < 0.001; ** p < 0.01; * p < 0.05.


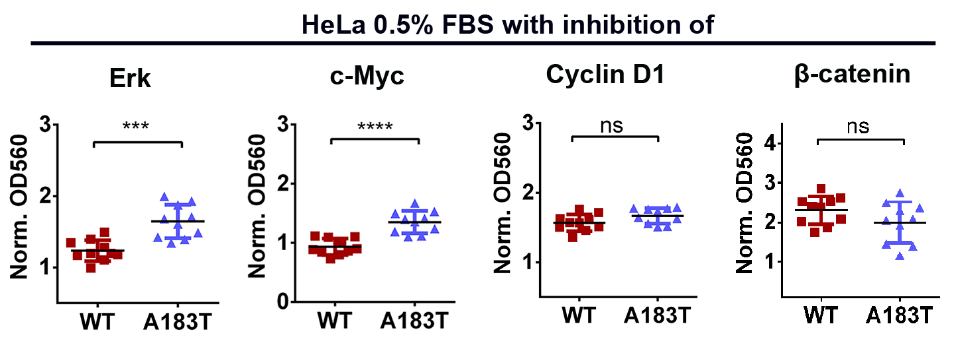


Supplementary Fig. 12 The normalized density of HeLa stable cells (WT or A183T) in the presence of 0.5% FBS and different inhibitors. Y-axis shows the cell density at 72 hours normalized by its density at 12 hours. PD98059 (50 µM), 10058-F4 (20 µM), Fascaplysin (0.1 µM), and Nitazoxanide (5 µM) are inhibitors against ERK, c-Myc, Cyclin D1, and β-catenin, respectively.
